# Laparoscopic Supracervical Hysterectomy with In-Bag Morcellation in Very Large Uterus

**DOI:** 10.1155/2017/9410571

**Published:** 2017-10-17

**Authors:** Harald Krentel, Rudy Leon De Wilde

**Affiliations:** ^1^Clinic of Obstetrics and Gynecology, St. Anna Hospital, Herne, Germany; ^2^Clinic of Gynecology, Obstetrics and Gynecological Oncology, University Hospital for Gynecology, Pius-Hospital Oldenburg, Medical Campus University of Oldenburg, Oldenburg, Germany

## Abstract

Laparoscopic supracervical hysterectomy (LASH) is a safe and fast minimally invasive approach in hysterectomy. In order to extract the uterine body from the abdominal cavity, one condition for LASH is the morcellation of the tissue. The intra-abdominal dissemination of benign and occult malignant uterine cells is a possible risk of this method, which can be avoided by the use of special bags for laparoscopic in-bag morcellation. We present a case of laparoscopic supracervical hysterectomy with in-bag morcellation in a uterus of more than 1400 g. and describe that this minimal-access surgery is safe and feasible even in very large uteri. This case report is registered in Research Registry under the UIN researchregistry1810.

## 1. Introduction

Laparoscopic supracervical hysterectomy (LASH) with electric power morcellation has become a popular minimally invasive approach in hysterectomy for benign pathologies in the last decade. Symptomatic uterine myomatosis or adenomyosis causing bleeding disorders or dysmenorrhea is the main indications for LASH. Tchartchian et al. showed the high satisfaction of patients undergoing LASH [1]. The rate of minor and major complications in LASH is very low [2]. Donnez et al. reported a major complication rate of 0.37% and a minor complication rate of 0.99% in 1613 cases [3], while Bojahr et al. described 0.3% major and 1.2% minor complications in 1706 cases [4]. Compared to total laparoscopic hysterectomy (TLH) a lower major complication rate in LASH has been described by Cipullo et al. [5]. Especially in large uteri the preparation of the bladder and the paracervical region is more difficult and a higher rate of complications can be expected in TLH [6]. In order to extract the uterine body from the abdominal cavity using the port incisions, the intraabdominal disrupture of the tissue is an inevitable condition of LASH. After a period of manual morcellation the development of modern electric power morcellators has led to a simple and fast morcellation process and made LASH a more attractive alternative. Morcellation related complications are rare, especially the direct intraoperative complications like vessel or bowel lesions [7]. But unfortunately with the extended use of the technique, the number of case reports about a new complication caused by intraabdominal cell dissemination during morcellation leading to parasitic peritoneal and retroperitoneal myomatosis, adenomyosis, and endometriosis after LASH increased in the last 10 years. Takeda et al. showed the identical histology of morcellated and disseminated tissue [8] and Miyake et al. reported the metastatic character of the spread tumors by molecular genetic analysis in 2009 [9]. The intraabdominal morcellation of occult malignant uterine tissue can cause an oncological upstaging combined with a potentially worsened prognosis of the malignant disease [10, 11]. After the first cases of intraabdominal morcellation of occult malignant uterine tumors the Food and Drug Administration warned against the use of electromechanical power morcellation in hysterectomy and myomectomy [12]. An overall rate of occult malignancy from 0.13% to 2.4% in laparoscopic supracervical hysterectomy has been described by various authors in retrospective studies. The use of recently developed bags for laparoscopic in-bag morcellation helps to minimize cell dispersal and could make LASH with laparoscopic intraabdominal morcellation a safer method preserving the benefits of minimally invasive surgery [13, 14]. However bag systems were initially criticized for their limitations in uterine size and weight. With this case report we describe the feasibility up to more than 1400 g.

## 2. Case

The 45-year-old patient presented with abnormal uterine bleeding and pelvic pain. Gynaecological examination revealed a very large uterus. In abdominal and transvaginal ultrasound we diagnosed uterus myomatosus with a total diameter of more than 20 cm. The uterine cervix did not show any pathological findings and a normal PAP smear within the last 12 month was documented. We indicated laparoscopic hysterectomy and the patient decided for LASH with in-bag morcellation after informed consent. We performed laparoscopic supracervical hysterectomy with in-bag morcellation using the More-Cell-Safe system (A.M.I., Austria) and the Rotocut electric power morcellator (KARL STORZ, Germany). The laparoscopic morcellation bag is a dual opening system, which allows two-port access with a protected optic against spread cell dissemination. The optical trocar was placed at the umbilicus with a 10 mm incision. The bag insertion and morcellation were performed through a 12 mm trocar in the left lower abdomen. In this case two additional auxiliary 5 mm trocars were needed due to severe adhesion after prior intraabdominal surgery. After complete laparoscopic adhesiolysis we performed the laparoscopic supracervical hysterectomy. After the dissection of the uterine cervix in the isthmocervical region with the monopolar hook, the uterine body was placed into the morcellation bag and in-bag morcellation was performed without any complications. There was no evidence of tissue or liquid spillage from the bag. Postsurgically we filled the bag with blue colored liquid and no bag lesion was obtained. The weight of the morcellated uterine body was 1420 g. The duration of the surgery was 175 min. The presurgical Hb was 9,7 mmol/l and the postsurgical Hb 9,2 mmol/l. The surgery was completed without any surgical or postsurgical complications. The histological examination revealed benign leiomyomas, no adenomyosis, and no malignancy.

## 3. Discussion

Benign peritoneal and retroperitoneal implants after LASH with intraabdominal morcellation can lead to symptomatic complications. Depending on the original morcellated uterine tissue, peritoneal myomatosis, adenomyosis, endometriosis, and endosalpingiosis have been reported. The parasitic fragments can cause pelvic pain, vaginal bleeding, and dysfunction of bladder, bowel, and ureter with subsequent secondary surgical interventions by laparoscopy or laparotomy. In a literature review of 44 publications, Meulen et al. identified sixty-nine women with parasitic myomas after laparoscopic morcellation. The overall risk for this complication was 0.12–0.95%. The median time between surgery and diagnosis was 48 months [15]. The risk of new peritoneal endometriosis after LASH with intraabdominal morcellation has been described to be 1.4% by Schuster et al. in a comparative study. They performed a second look laparoscopy in 12 symptomatic patients, but not in all patients who underwent LASH in this study [16]. Possibly more than 12 patients had a new iatrogenic peritoneal endometriosis but so far without symptoms. Donnez et al. examined more than 1400 patients after LASH and reported intraabdominal tumors in 0.57% of all cases [17]. All these 8 patients presented adenomyosis in the initial histology of the uterine corpus. It would be interesting to relate these patients to the group of all patients with initial adenomyosis in this study. Especially as symptomatic adenomyosis is a main indication for LASH, it would be helpful to know the true risk rate of iatrogenic peritoneal endometriosis and adenomyosis after LASH with electronic power morcellation. Various authors described the risk rate for unexpected malignancy in presumed benign conditions in laparoscopic supracervical hysterectomy and myomectomy by retrospective analysis. The overall risk of unexpected malignant uterine tumors in LASH ranged from 0,13% in 10731 cases [7] to 2,4% in 778 cases [18]. Picerno et al. recently described a rate of 0,8% of occult malignant and premalignant uterine pathologies in a retrospective cohort study [19] and an overall risk of 0.41% of malignancy in 734 morcellated surgical specimen after laparoscopic hysterectomy and myomectomy has been reported by Tan et al. [20]. Some authors described presurgical diagnostic methods in order to detect occult malignancy. A presurgical PAP smear and transvaginal ultrasound should be standard procedures and diagnostic hysteroscopy or abrasion prior to LASH might help to diagnose endometrial malignant or premalignant submucous lesions. However, rare intrauterine malignant mesenchymal tumors could be misdiagnosed as benign lesions in hysteroscopy [21]. Regarding this presurgical procedures the overall risk rate of 0.25% of unexpected malignancy in 1584 cases stays within the reported range [22]. Sarcomas especially can not be safely distinguished from myomas. In this discussion the difficulties in the histopathological examination in morcellated tissue have to be considered. In 1997 Schneider described a case of initially unremarkable tissue evaluation after LASH with morcellation. But the retrospective histopathologic examination after 5 months showed cluster of malignant cells [23]. Rivard et al. reported a prospective case series that demonstrated the problems in detection and staging of uterine cancer in morcellated tissue. Five uterine specimens with benign lesions and five specimens with endometrial cancer were morcellated after initial pathologic analysis. In the morcellated specimens 20% of the malignancies were not detected and oncologic staging was not possible in all cases [24]. In order to aid the pathologist in identifying the endometrial layer Tam et al. described a technique to stain the endometrium before morcellation [25]. In addition benign disseminated uterine tissue can transform to premalignant and malignant tumors years after the initial surgery. This might be a long-term complication with a so far unknown risk rate. The progression of a lost uterine tissue fragment after morcellation to peritoneal complex atypical endometrial hyperplasia has been reported in 2011 [26]. Various authors described the possible malignant transformation of endometriosis and adenomyosis [27–29]. Since the FDA warning against the use of intraabdominal electric power morcellation in hysterectomy and myomectomy the method has been under permanent discussion. As a first consequence the risk of benign or malignant cell dissemination during morcellation had to be discussed with the patients as part of the informed consent. Most authors stated that the morcellation process always should be followed by meticulous sampling of lost fragments and peritoneal lavage. Unfortunately the above-mentioned risks can not be avoided sufficiently that way. Alternative minimally invasive approaches in large uteri like total laparoscopic hysterectomy or laparoscopically assisted vaginal hysterectomy also require the transvaginal morcellation of the tissue when feasible. An alternative to inevitable intraperitoneal cell dissemination, subsequent sampling and lavage is the use of morcellation bags. Boruta and Shibley recently reported a case of contained morcellation of an unsuspected uterine leiomyosarcoma [30]. They conclude that the use of in-bag morcellation may minimize the risk that women have a worse prognosis when unexpected malignancy is diagnosed after laparoscopic hysterectomy with morcellation. Ikhena et al. reported about intraabdominal washings before and after laparoscopic in-bag morcellation and found no cytologic evidence of intraabdominal cell dissemination after enclosed morcellation [31]. Contrarily a rate of 9.2% of liquid or tissue spillage after contained power morcellation was reported by Cohen et al. in a multicenter prospective cohort study in 76 patients. Although containment bags were intact in all cases, applied blue dye leakage was found [32]. Rimbach et al. reported negative peritoneal washings for muscle cells in all cases of bag use in laparoscopic hysterectomy in a pig model, while positive cytology was found in 5 out of 8 cases in open power morcellation [33]. They subsequently described the first clinical human experiences with the More-Cell-Safe system. The time associated with the bag use ranged from 8.5 to 26.5 min. The morcellated specimen weight ranged from 205 to 638 g. [34]. Anapolski et al. described the use of a containment bag in ten cases of laparoscopic supracervical hysterectomy. They reported a mean time for bag insertion and preparation of 10.5 min and a mean morcellation time of 10.5 min. The mean specimen ranged from 32 to 710 g. [35]. A different technique for laparoscopic in-bag morcellation was described by Aoki et al. The mean bag introduction time was 21.8 min; the mean in-bag morcellation time was 11.5 min in 12 patients undergoing laparoscopic hysterectomy and myomectomy. They reported no bag damage [36]. In comparison Solima et al. reported a rate of 33% bag lesions after vaginal in-bag morcellation during total laparoscopic hysterectomy [37]. Especially in large uteri the risk of bag lesions seems to be higher. In case of tissue or liquid spillage from the bag the fragments should be removed from the abdomen combined with laparoscopic lavage. The impact of bag lesions on the risk rate of intraabdominal cell dispersal or the eventual upstaging of occult malignant tumors has to be evaluated in future studies. The use of laparoscopic in-bag morcellation is limited to a certain specimen size depending on the used system. McKenna et al. reported in-bag morcellation in a uterine weight of 978 g. [38]. In a multicenter study with 73 patients Cohen et al. reported a case of contained power morcellation within an insufflated bag in a uterine weight of 1481 g. [39]. However so far the uterine size and weight represented an important limitation for the use of morcellation bags. In this case we realized the laparoscopic supracervical hysterectomy with laparoscopic in-bag morcellation through a 12 mm port in a uterine weight of 1420 g. Usually we realize this procedure with a 10 mm optical trocar, a 12 mm auxiliary trocar, and a 5 mm auxiliary trocar. This shows that the laparoscopic contained morcellation technique is a safe and feasible option even in very large uteri preserving the advantages of minimal-access hysterectomy. The use of morcellation bag systems is correlated with an additional operative time [40, 41], which ranges from approximately 8.5 to 30 min. The time loss depends on the experience of the surgeon, the specimen size, and the bag system. General conditions as patients weight and positioning of auxiliary trocars also have an effect on the duration of the procedure. However, the surgical time for tissue sampling after uncontained morcellation can be saved using the in-bag technique. Different techniques of contained scalpel or manual morcellation and vaginal morcellation techniques have been recently described. In laparoscopic supracervical hysterectomy the vaginal approach is not feasible. External manual or scalpel techniques are combined with larger incisions in order to extract the uterine tissue. The laparoscopic in-bag morcellation through a 12 mm port preserves the minimally invasive approach.

## 4. Conclusion

The use of in-bag morcellation minimizes the risk of inadvertent tissue dissemination in occult malignancy or benign conditions like adenomyosis or leiomyomatosis. Laparoscopic supracervical hysterectomy with laparoscopic in-bag morcellation is a safe and feasible minimal-access surgery even in very large uteri of more than 1400 g. Thus laparoscopic supracervical hysterectomy with contained power morcellation can be offered to the patients as a safe alternative approach with all benefits of laparoscopic surgery and abdominal hysterectomies being avoided.
